# 

**Published:** 1890-01

**Authors:** 


### LIST OF CONTRIBUTORS.

Baird, William.
Berns, George H.
Bower, George F.
Breisacher, Leo.
Brimhall, S. D.
Bryce, Peter H.
Bryden, Williamson.
Butler, G. W.
Coates, J.
Conklin, W. A.
Fleming, George.
Frink, J. H.
Frothingham, Langdon.
Gimbert, Dr.
Griffin, G. E.
Hatch, J. Leffingwell.
Hinebauch, T. D.
Huidekoper, R. S.
Hutcheon, D.
Jeffries, John Amory.
Lawley, E. S.
Lawley, E. T.
Lee, Daniel D.
Liautard, Prof. A.
Linsley, Jo H.
Lyford, C. C.
Marcy, Henry O.
Martin, William G.
Mason, R. C.
McCall, Prof. James.
McLaughlin, John A.
Meyers, J. C.
Morris, Claude D.
Paquin, Paul.
Parker, John M.
Ridge, W. H.
Salmon, D. E.
Schwartzkopff, Olof.
Shufeldt, R. W.
Sibley, Walter K.
Smith, Theobald.
Stewart, S.
Sutton, J. Bland.
Thomassen, Prof.
Trasbot, Prof. L.
Waugh, James A.
Welch, Wm. H.
Williams, W. L.

136430

### INDEX VOLUME XI.

Age of the Horse, Ox, Dog, and other Domesticated Animals, 32, 107, 167, 237, 279, 340, 390, 455, 493, 578, 608, 702
Alumni Association of the American Veterinary College.................... 256
American Veterinary College ............................................ 258, 528
Animal Suture ; Its Place in Surgery............................................ 215
Anomaly, Molar Tooth.......................................................... 463
Antiseptic Surgery............................................................... 267
Army Veterinary Service........................................................ 12
Autopsy on the Central Park Chimpanzee ; Report of the.................. 229
Avian Anatomy.................................................................. 22

Baird William. Remounts, United States Army.............................. 132
Baltimore School of Veterinary Science................................. 528, 675
Berns, George H. Moss as a Substiture for Linseed Meal and Hot Woolen Cloths.................................................. 575
Osteo-porosis .................................................................. 714
Books and Pamphlets Received. 88, 142, 195, 260, 315, 365, 413, 482, 534, 680
Bower, George F., Practical Hints ............................................ 276
Breisacher, Leo, Experiments upon the Superior Laryngeal Nerve....... 69
Brimhall, S. D., Lympho-Sarcoma.............................................. 234
Bubonocele in Horse ........................................................ 719
Bryce, Peter H., Contagious Venereal Diseases Amongst Horses in Kent County, Ontario ................................................... 143, 197
Bryden, Williamson, Fibroid Tumors of Shoulder............................ 236
The Horse's Hoof.............................................................. 334
Bubonocele in Horse ........................................................... 719
Butler, G. W., Rabies and Strongylus Tetracanthus as a Coincidence in the Horse.................................................................... 433

Central Park Menagerie, Autopsy Kangaroo.................................. 64
Chicago Veterinary College................................................ 312, 529
Coates, J., Glycerine in Veterinary Practice ................................ 118
Comparative Psycology, Proceedings of the Society for the Study of..... 71
Conklin, W. A., Breeding of a Hippopotamus.................................. 30
Contraction of the Horse's Foot............................................... 553
Contagious Pleuro-Pneumonia in Goats at Cape Colony ..................... 183
Venereal Diseases Amongst Horses in Kent County, Ont., 143, 197
Review of ..................... 261
Correction, A....................................................................... 400
Cotton-Seed Cakes ................................................................ 571

Dehorning Cattle............................................................... 513, 516
Detention of Mexican Hairless Dogs, Notes on the............................. 235
Diseases of the Domesticated Animals, Some Recent Researches in the. 535

Editorial Note........ 596
EDITORIAL.—Dr. Huidekoper Withdraws from the Veterinary Department, University of Pennsylvania........ 59
Joint Meeting of the Illinois and Indiana Veterinary Medical Association........ 350
Molar Extractor and Cutter........ 350
Number and Values of Farm Animals in the United States........ 243
Paper Horse Shoes........ 290
Pennsylvania State Veterinary Medical Association........ 513
Recognition of American Veterinarians........ 242
THE JOURNAL OF COMPARATIVE MEDICINE AND VETERINARY ARCHIVES........ 57
Tuberculosis........ 115
Typographical Errors........ 117
United States Army Veterinary Notes........ 466
Veterinary Medical Association... 117, 178, 289, 347, 466, 512
Veterinary Journalism........ 464
Veterinary Legislation........ 113
Veterinary Progress a Gain to Science........ 174
Entropion in a Dog........ 164
Etymology of the Word "Veterinary"........ 412

Faculty of Comparative Medicine and Veterinary Science..........83, 306, 528
Fleming, George, Physical Condition of Horses for Military Purposes... 120
Frink, J H., Serous Cyst in Superior Maxillary Sinus........ 491
Frothingham, Langdon, A Case of Lupus, or Cutaneous Tuberculosis.... 103

Gastrotomy........ 173
Gimbert, Dr., Vertiginous Symptoms Consecutive to Clipping in the Horse........ 65
Glanders Bacillus, On the Influence of Slight Modifications of Culture Media on the Growth of Bacteria as Illustrated by the........ 158
Glycerine in Veterinary Practice........ 118
Griffin, G. E., Entropion in a Dog........ 164
Malarial Fever in Horses........ 696

Hæmatoma, Recurrent Tumor in the Ox........ 67
In a Cow........ 68
Harvard University, Veterinary Department........ 412
Hatch, J., Leffingwell, Some Original Investigations upon the Python Molurus........ 415
Heart Disease in the Horse........ 165
Hinebauch, T. D., The Wormian and (?) Bones........ 597
Hints to Explorers and Naturalists in the Field, About the Preparation, Care and Transportation of Vertebrate Skeleton in the Rough........ 98
Hippopotamus, Breeding of........ 30

Hoof, the Horse's........ 334
Horse Show Aseociation of America, National........ 676
Huidekoper, R. S., Army Veterinary Service........ 12
Age of the Horse, Ox, Dog and Other Domesticated Animals, 32, 107, 167, 237, 279, 340, 390, 455, 493, 578, 608, 702
Contraction of the Horse's Foot........ 553
Hutcheon, D., Contagious Pleuro-Pneumonia in Goats at Cape Colony... 183
Hydrops Uteri as a Sequel to Imperforate Vagina........ 510

Illinois State Veterinary Medical Association........75, 249, 676
Indiana Association of Veterinary Graduates........ 189
Iodide of Potash, The Action of........ 246
Iowa State Veterinary Medical Association........676, 724

Jeffries, John Amory, Etiology of two Outbreaks of Disease among Hogs 681
Joint Meeting of the Illinois and Indiana Veterinary Medical Associations 350
JOURNAL OF COMPARATIVE MEDICINE AND VETERINARY ARCHIVES... 57

Kangaroo, Autopsy of........ 64
Kansas Veterinary Medical Society........401, 726
Keystone Veterinary Medical Association..80, 138, 192, 255, 300, 362, 402, 587, 727
Koch's Treatment for Tuberculosis........ 720

Lawley, E. S., Gastrotomy........ 173
E T., Antiseptic Surgery........ 267
Lee, Daniel D., Six Cases of Tumors........ 27
Legal Decision........ 399
Liautard, Prof. A., Veterinary Jurisprudence........ 543
Linsley, Jo H., Report of the Autopsy on the Central Park Chimpanzee. 229
Long Island Veterinary Society........193, 253, 291, 364, 411, 726
Lupus, or Cutaneous Tuberculosis, A Case of........ 103
Lyford, C. C., Contagiousness of Tuberculosis........ 570
Lympho-Sarcoma........ 234

Mad Itch........ 386
Malarial Fever in Horses........ 696
Marcy, Henry O., Animal Suture; its Place in Surgery........ 215
Martin, William G., Molar Tooth Anomaly........ 463
Hydrops Uteri as a Sequel to Imperforate Vagina.... 511
Massachusetts Veterinary Association........403, 466, 726
Mason, R. C., Sarcoma........ 106
McCall, Prof. James........ 194
McLaughlin, John A., Heart Disease in the Horse........ 165
Meat Inspection, National and International........ 599
Meyers, J. C., Cotton-Seed Cakes........ 571
Minnesota, University of........ 675
Molar Extractor and Cutter........ 350

Montreal Veterinary Medical Asssociation........................ ................. 301
Morris, Claude D., Tympanitis Cured with Carbolic Acid ..................... 398
Moss as a Substitute for Linseed Meal and Hot Woolen Cloths............... 575

Nariman, Dr., Hæmatoma—Recurrent Tumors in the Ox .................... 67
Necrology—James Brodie.............................................. .................. 84
Charles C. Moulton............................................................... 84
E. F. Thayer........................... ........................................ 84
Alexander Lockhart ................ ......................................... 140
Armand Charles Goubaux................................................ 479
William Raeburn Jenkins...... .......................................... 524
Nerve, Experiments upon the Superior Laryngeal............................... 69
New Jersey State Veterinary Society................................................... 77
Veterinary Medical Association ...............................79, 298, 527
New York College of Veterinary Surgeons............. ...................258, 528, 675
State Veterinary Medical Society...................................190, 526
Northwestern Veterinary College........................................................ 83
Notice..................... .......... ............................................................. 400
Number and Values of Farm Animals in the United States.................. 243

Odontomes ..................................................................... ........ ..................1, 89
Ohio, University of the State of... ........................................................ 476
State Veterinary Medical Association....................................188, 525
Ontario College, Veterinary Medical Society of the............................ 299
Ontario Veterinary College. ..........................................139, 194, 302, 727
Open Letter............................................................................476, 530
Osteo-porosis........................................................................................ 714

Paper Horse Shoes.................................................................................. 290
Paquin, Paul, Texas Fever ................................................367, 436, 710
Parker, John M., Tumors of Spinal Cord.................................................. 384
Parturient Fever of the Cow, a Contribution to the Study of................. 351
Pathology in its Relations to General Biology ...................................... 179
Pennsylvania State Veterinary Medical Association.........................513, 583
Veterinary Society (Franklin Co.)................................. 529
Physical Condition of Horses for Military Purposes.............................. 120
Pig Feeding............................................................................................. 346

Practical Hints........................................................................................ 276
Python Molurus, Some Original Investigations upon the..................... 415

Rabies and Strongylus Tetracanthus as a Coincidence in the Horse...... 483
Recognition of American Veterinarians................................................ 242
Rectum, Prolapsus and Amputation....................................................... 462
Remounts, United States Army.............................................................. 132
Resistance to Hydrocyanic Acid Poisoning ........................................... 398
Review Department.............................86, 141, 195, 313, 480, 533, 677, 730
Ridge, W. H., Hæmatoma in a Cow........................................................ 68
Roaring, The Operative Treatment of ............. ................................... 521

Salmon, D. E., The Recent Review of Swine Disease ........................ 41
Sarcoma.................................................................................. 106
Schwartzkopff, Olof, National and International Meat Inspection......... 599
Serous Cyst in Superior Maxillary Sinus............................................ 491
Shufeldt, R. W., Progress in Avian Anatomy..................................... 22
Hints to Explorers and Naturalists in the Field, about the Preparation, Care and Transportation of Vertebrate Skeletons in the Rough.................................... 98
Sibley, Walter K., Tuberculosis in Birds ........................................... 317
Smith, Theobald, Influence of Culture Media on Growth of Bacteria ... 159
Stewart, S., Mad Itch ........................................................................ 386
Sutton, J. Bland, Odontomes. .......................................................1, 89
Swine Disease Literature, Recent Review of....................................... 41
Etiology of Two Outbreaks Among Hogs ..................... 683

Texas Fever..........................................................................367, 436, 710
Thomassen, Prof., A Contribution to the Study of Parturient Fever of the Cow ....................................................................................... 351
Trasbot, Prof. L., The Action of Iodide of Potash............................. 246
Tuberculosis, Contagiousness of........................................................ 570
Tuberculosis in Birds......................................................................... 317
Tuberculosis ...................................................................................... 115
Present Attitude of Veterinarians on the Subject....... ... .. 162
Tumors of Shoulder, Fibroid .............................................................. 236
Six Cases of ........................................................................ 27
of Spinal Cord... ................................................................. 384
Tympanitis Cured with Carbolic Acid................................................. 398
Typographical Errors ......................................................................... 117

United States Veterinary Medical Association, ...117, 178, 289, 347, 466, 512
592, 613, 723
University of Pennsylvania, Veterinary Society of..........................82, 301
United States Army Notes.................................................................. 466

Vertiginous Symptoms Consecutive to Clipping in the Horse.............. 65
Veterinary Department, University of Pennsylvania.......................... 365
Journalism.................................................................... 465
Jurisprudence.........................................................516, 543
Legislature .................................................................... 113
Progress a Gain to Science............................................. 175

Waugh, James A., Notes on the Dentition of Mexican Hairless Dogs.... 235
Prolapsus and Amputation of Rectum ..................... 462
Welch, Wm. H., Pathology in Its Relations to General Biology ........... 179
Williams, W. L., Review of Contagious Venereal Disease Amongst Horses .............................................................................................. 261
Wormian and — ? — Bones................................................................ 597

THE United States Medical Association has issued a revised copy of their constitution and by-laws, stating the aims and objects of the association, with the by-laws governing the members. Any member of the profession desirous of joining the association can secure a copy of the same by addressing the secretary, W. Horace Hoskins, 12 South 37th street, Philadelphia, Pa.

---

COMMUNICATIONS concerning peculiarities and abnormalities of dention and facts of special interest in regard to the age of our domesticated animals, will be acknowledged and used in the serial artitcle on this subject commencing in the present number.

---

A NUMBER OF valuable communications have been received, which must be delayed until future issues for want of space.

of dealing with this interesting feature of his work is very clear and logical, and lends atiraction to a deep and difficult question. He is not only a shrewd observer, but an exact thinker, and it is evident from the many ingenious reflections and casual remarks with which his book abounds, that he possesses a logical and well trained mind.

C. M. O'L.

### BOOKS AND PAMPHLETS RECEIVED.

"Cow's Milk for Infant Food," by E. F. Brush, M. D.

"Urinary Calculous and Lithotomy," by Thomas W. Kay, M.D. Reprinted from Maryland *Med. Jour.*

"Eleventh Annual Report of the State Board of Health of Rhode Island.' Providence, 1889.

"Sechsundsechzigster jahres-bericht der Schleischeu Gesellschaft fur vaterlandische Cultur." Breslau, 1889,

"Journal of the Natural History Society." Edited by H. M. Phipson, Hon. Secretary. Vol IV., No. 2. Bombay, 1889.

"Proceedings of the Royal Society of Queensland." Vol., Part V.

"Journal of Cincinnati Society of Natural History." Vol. XII., Nos. 2 and 3.

"Bulletin de L'Academie Royal de Medicine de Belgique." T. III., Nos. 8–9. Brussels, 1889.

"Archives Neerlandaises des Sciences Exactes et Naturalles." Edited by J. Bosscha. T. XXII. Liv. 5. Harlem, 1889.

"Sitzungsbericht der Math-phys. Classe der K. B. Akademie der Wissenschatten." 1888, Heft III., 1889, Heft I–II. Munich, 1889.

"Bulletin Societé Zoologique de France." October. Paris, 1899.

"Studies of the Macrochires, morphological and otherwise, with a view of indicating their relationships and defining their several Positions in the System." By R. Shufeldt, M.D., C.M.Z.S., Capt. Med. Corps., U. S. A. Reprinted from the *Linnean Society's Jour. Zoology.* Vol. XX.

"Contributions to the Comparative Osteology of the Families of North American Passeres and Notes on the Anatomy of Speotyto Curricularia Hypogæa." By R. W. Shufeldt, M.D. Reprinted from the *Journal of Morphology*, Vol. III., No. 1.

"Report of the U. S. Commission of Fish and Fisheries for 1866." Washington, 1889.

"North American Fauna," Nos. 1 and 2, by C. Hart Merriam, M.D. U. S. Dept. Agriculture. Washington, 1889.

"Ornis. Internationale Zeitschrift fur die gesaammite Ornithologie.'. Jah. V. Heft III. Vienna, 1889.

"Le Naturaliste Canadien." Vol. XIA., Nos. 4 and 5. Cape Rouge, 1889.

"Il Naturalista Siciliano—Giornale de Scienze Naturali," Palermo, 1889.

